# Implementing a digital public health project - lessons learned? Stefan Buttigieg

**DOI:** 10.1093/eurpub/ckac129.151

**Published:** 2022-10-25

**Authors:** S Buttigieg, H Agius Muscat, J-P Ebejer

**Affiliations:** 1 Ministry for Health, Valletta, Malta; 2 EUPHA-DH; 3 Digital Health Malta, Valletta, Malta; 4 University of Malta, Valletta, Malta

## Abstract

The demand for the digitalisation of public health has been ongoing for more than a decade. The COVID-19 pandemic was the tipping point that accelerated the ideation, implementation, and scale-up of such public health projects. Despite the well-needed push, the same challenges that face every similar implementation will nonetheless be the same if not accentuated. The scope of the presentation is to highlight the difficulties and facilitators that such implementations and evaluations bring forward. We will also see what we can learn as public health professionals to ensure that present and future information systems are well-planned. We have to ensure that they do not succumb to the pressure of well-intended stakeholders who are yearning for such solutions to help their business workflows. This presentation will be enhanced with the lessons learned from implementing, monitoring, and following up on Malta's national contact tracing app and Customer Relationship Management systems based on Microsoft Dynamics 365 technologies that tackled the Test, Track and Trace workflows that were integral to the COVID response in Malta.

